# A Rare Case of Multifocal Asynchronous Benign Granular Cell Tumors with *PIK3CA* Subclonal Mutation Identified in One Tumor by Next-Generation Sequencing

**DOI:** 10.1155/2023/2932512

**Published:** 2023-01-24

**Authors:** Tiago Palmisano, Tina Bocker Edmonston, Thomas Holdbrook, Shuyue Ren

**Affiliations:** ^1^Cooper Medical School of Rowan University, 401 Broadway, Camden, New Jersey, USA 08103; ^2^Department of Pathology and Laboratory Medicine, Cooper University Hospital, 1 Cooper Plaza, Camden, New Jersey, USA 08103

## Abstract

Granular cell tumor (GCT) is a benign neuroectodermal tumor typically in the dermis or subcutis, although deep soft tissues and organs are occasionally involved. Multifocal GCTs are estimated to occur as many as 10% of patients. A 40-year-old female presented with multiple GCTs asynchronously involving various body sites including gastrointestinal, gynecologic, breast, urinary, and soft tissue systems. Pathologic examinations suggested benign GCTs. TruSight Tumor 170 next-generation sequencing (NGS) analysis performed on four resected tumors revealed subclonal mutation of *PIK3CA* p.H1047R identified in the esophageal GCT but not in the right vulva or the two cecal GCTs, suggesting that each is a primary tumor with a distinct genetic profile, rather than metastasis. *PIK3CA* p.H1047R is a common mutation in many cancers. Our benign GCT case demonstrates *PIK3CA* mutation with a low mutant allele frequency of 7%, which may represent an evolving subclone and might confer a more aggressive behavior.

## 1. Introduction

Granular cell tumor (GCT) is a rare soft tissue neoplasm with evidences thought to be derived from neural/Schwann cells. Recently, endomesenchymal origin has been suggested; however, the pathogenesis is still uncertain [1–9]. The majority of GCTs are benign, and malignant GGTs are extremely rare. Most cases affect deep dermis and subcutaneous tissue, and other sites include the tongue, breast, gastrointestinal tract, respiratory tract, and genital system. Although GCTs are mostly solitary, as many as 10% are multifocal, which can be regional or involve multiple body sites [10, 11]. Multiple GCTs have been reported in association with various syndromes [12], such as neurofibromatosis type I, Noonan syndrome, LEAPARD syndrome, and Watson syndrome. The tumors are painless, hard, irregular nodules typically less than a few centimeters in diameter with ill-defined margins. Histologically, GCT is composed of large monotonous epithelioid cells with intensely eosinophilic, granular cytoplasm [3]. The Fanburg-Smith classification, used worldwide for GCTs, subdivides the tumor into malignant, atypical, or benign according to the presence or absence of the following six histologic features: nuclear pleomorphism, tumor cell spindling, vesicular nuclei with large nucleoli, increased nuclear cytoplasm ratio, necrosis, and increased mitotic rate (>2 mitoses/10 high-power fields). Tumors are classified as malignant if there are 3 or more of these findings [1]. When GCTs are located dermally, they can present with pseudoepitheliomatous hyperplasia of the overlying skin, and if extensive, that can be mistaken for squamous cell carcinoma. Here, we describe a case of asynchronous multiple body foci of GCTs. TruSight Tumor 170 comprehensive NGS assay that targets DNA and RNA variants covering 170 genes associated with solid tumors was performed on four of the resected tumors to search for the possible pathogenesis. One of the tumors, the esophageal tumor, had a *PIK3CA* p.H1047R mutation present in 7% of the reads.

## 2. Case Summary

The patient is a 40-year-old African American female with a 27 pack-year smoking history who first presented in 2014 with two perianal lesions that the patient claimed had started small but had been growing since 2011. Excisional biopsy revealed these lesions to be GCTs. Two years later, the patient returned with five vulvovaginal subcutaneous lesions, 1-2 cm in size, which were excised and confirmed to be GCTs. In 2018, the patient underwent a barium swallow and colonoscopy due to laryngopharyngeal reflux and hematochezia, which demonstrated a mediastinal soft tissue mass and a 0.7 cm GCT in the cecum. A follow-up CT scan of the mediastinum confirmed a 2 cm mass on the cervical esophagus, and endoscopic fine-needle aspiration was diagnostic for GCT, which was subsequently excised along with a left parathyroid adenoma. That same year, the patient had an MRI for migraine headaches, which demonstrated a left parietal cavernous hemangioma. In 2020, the patient presented with multiple new vulvovaginal and rectal nodules, and a pelvic MRI showed multiple enhancing foci in the vulva, vagina, cervix, breast, urinary bladder, left and right gluteus maximus muscles, left tensor fascia lata muscle, and perianal soft tissue, all of which were suspected to be granular cell tumors. In August 2020, a right medial thigh mass resection performed at an outside hospital revealed GCT. In April 2021, right breast and right axillary tail biopsies performed at an outside hospital revealed three foci of GCTs. There is no known history of genetic conditions for this patient.

### 2.1. Histological and Immunohistochemical Findings

**Figure 1:**
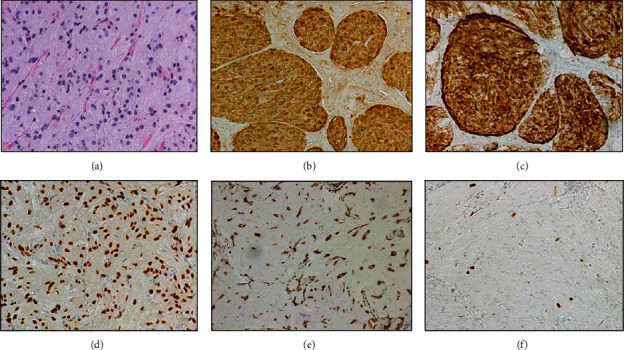
Granular cell tumor. Histological sections of the tumor with H&E stain (a) and immunohistochemical stains positive for S-100 (b), inhibin (c), and SOX10 (d), negative for CD163 (e), and low Ki-67 proliferative index (f). All ×200.

The excision and diagnostic studies of the perianal lesions from 2014 were performed at an outside institution, where the pathology data was unavailable. Excision of the five subcutaneous vulvovaginal lesions in 2016 revealed irregularly shaped, pale-tan, solid nodules involving fibrofatty tissue. The nodules had a maximum diameter of less than 2 cm. The tumors were composed of sheets of large monotonous epithelioid to polygonal cells with intensely eosinophilic, granular cytoplasm, and indistinct cell borders. Nuclei are centrally located ranging from small to larger and vesicular with distinct nucleoli. Mitoses were not prominent and necrosis was not noted. The morphological features together with a positive S-100 immunohistochemical stain confirmed the diagnosis of GCT. Sectioning of the excised cecal polyp in 2018 showed a submucosal well-circumscribed pale-tan whorled 0.8 × 0.8 × 0.7 cm nodule with the same morphological features of GCT. Immunohistochemical stains were positive for S-100 and inhibin and were negative for CD163, CD34, desmin, DOG-1, CD117, and smooth muscle actin. Based on these studies, the cecal nodule was diagnosed as GCT. Biopsy of a submucosal mass in the same location was obtained on a follow-up colonoscopy two years later. The mass was tan, ovoid, and measured 1.0 × 0.5 × 0.2 cm. Immunohistochemical stains were once again positive for S-100, inhibin, and SOX10 and negative for CD34, desmin, DOG-1, CD117, smooth muscle actin, and pancytokeratin. The Ki-67 proliferative index of this recurrent tumor was less than 2% (Figure 1). Immunohistochemical stains of the fine-needle aspirate from the esophageal mass in 2018 also confirmed the diagnosis of GCT, with a Ki-67 proliferative index of 1%. Excision of the esophageal masses revealed three irregularly shaped, pale-tan nodules ranging from 0.5 to 1.5 cm in diameter. Histological sections revealed the same morphological features and immunohistochemical profile of GCT. No features suggestive of malignancy were identified in the above GCTs.

### 2.2. NGS Findings

**Figure 2:**
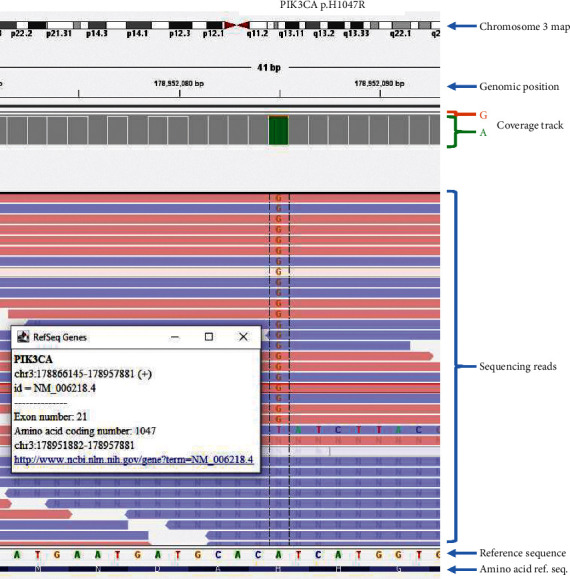
*PIK3CA* p.H1047R. Alignment in IGV_2.12.0 (integrative genomics viewer, https://software.broadinstitute.org/software/igv) shows a substitution c.3140A>G with 124 reads of G (orange) and 1640 reads of A (green), schematically shown in the column diagram of the coverage track. This changes the triplet code CAT for Histidine (H in the amino acid reference sequence) to CGT for Arginine in codon 1047 in exon 21 of the *PIK3CA* oncogene (reference sequence: NM_006218.4).

To further characterize the tumors, TruSight Tumor 170 comprehensive NGS assay, which targets DNA and RNA variants covering 170 genes associated with solid tumors, was performed on the esophageal, right vulva, and cecal tumors. No gene sequence variants were identified in the reported associated syndromes' related genes including NF1, PTPN11, KRAS, BRAF, and MAP2K1. However, the esophageal tumor had a *PIK3CA* p.H1047R mutation that was pathogenic but only present in 7% of the reads (Figure 2). The lack of symptoms associated with multifocal lesions, along with multiple biopsies that lacked signs of malignancy, led her physicians to opt for an observational approach rather than attempt further excisions.

## 3. Discussion

GCTs are typically less than a few centimeters, painless nodules. Histologically, GCT is composed of sheets, nests, and trabeculae of large monotonous epithelioid to polygonal cells with intensely eosinophilic, granular cytoplasm. Cell borders may be indistinct, producing a syncytial appearance. Nuclei are usually centrally located ranging from uniformly small and mildly hyperchromatic to larger and vesicular with distinct nucleoli. Mitoses are variable in number but usually not prominent. The finely granular appearance of the cytoplasm is strongly periodic acid-Schiff- (PAS-) positive and diastase-resistant, and the granules resemble lysosomes ultrastructurally [3]. The tumors express the immunohistochemical markers S100, SOX10, vimentin, CD56, NSE, CD63, CD68, and inhibin [13–16].

The patient has multiple GCTs involving at least six body sites including the esophagus, breast, thigh, cecum, perianus, and vulva. All tumors showed similar morphological features, lacking atypical or malignant features, and were diagnosed as benign GGTs. The major differential diagnosis of GGT includes melanoma, alveolar soft tissue sarcoma, malignant peripheral nerve sheath tumor, and gastrointestinal stromal tumor. However, the diagnosis of GCT, with characteristic morphological features and immunohistochemical profile, is usually not challenging. Many reports suggested that multiple GGTs are associated with some syndromes such as LEOPARD syndrome, neurofibromatosis, Noonan syndrome, and Watson syndrome; however, to date, no genetic testing was performed in this patient. TruSight Tumor 170 comprehensive NGS assay was performed on the patient's resected esophageal, right vulva, and cecal tumors. The esophageal tumor had a *PIK3CA* p.H1047R mutation that was present in 7% of the reads, which could represent a subclonal somatic mutation. There were no amplifications or fusions noted in the four tumors. No gene sequence variants were identified in associated syndromes' related genes including NF1, PTPN11, KRAS, BRAF, and MAP2K1. Unfortunately, ATPase genes are not built into the TruSight Tumor 170 comprehensive NGS panel and the tumor's ATPase status is unknown.

Loss-of-function mutations affecting the vacuolar H+-ATPase (V-ATPase) accessory genes, ATP6AP1 and ATP6AP2, were found in 72% of the granular cell tumors collectively. A loss-of-function mutation was detected in BRD7 (bromodomain-containing 7), a recently recognized tumor suppression candidate. Dehner et al. recently demonstrated that multifocal GCTs frequently harbor inactivating mutations in V-ATPase genes, most commonly ATP6AP1 and ATP6AP2. Further, multifocal GCTs within a given patient were molecularly distinct, supporting interpretation as separate primaries. In contrast, paired primary and metastatic histologically malignant GCTs showed identical mutations, confirming the primary-metastatic relationship [17]. Wei et al. [18] reported whole exome sequencing (WES) performed in a malignant GCT that exhibited low mutation burden and overall stable genome with local complex rearrangements. In the 2015 USCAP meeting [19], Bavi et al. performed WES on benign and malignant GCTs and only malignant GCT showed alterations in key known driver oncogenes such as *TP53* and *PIK3CA*. No detailed genetic alterations were shown in the abstract. Two cases of GCTs of thyroid were reported recently, one case demonstrated a clonal ATP6AP1 p.G381Vfs∗15 frameshift mutation. The second case identified a clonal ATP6AP2 p.L182Pfs∗22 frameshift mutation along with a *PIK3CA* H1047R hotspot mutation [20].

*PIK3CA* is among the most commonly mutated genes in cancer. The *PIK3CA* gene encodes the catalytic subunit of phosphatidylinositol-3 kinase (PI3K). A variety of receptor tyrosine kinases such as EGFR, ERBB2 (HER2), RET, MET, and VEGFR activate PI3K. PI3K then triggers intracellular downstream AKT/mTOR signaling that promotes cell survival, proliferation, growth, and motility [21]. Activating mutations of this oncogene are seen in 30% to 40% of the endometrial cancers and breast cancers as well as in many other malignancies [21–24].

In summary, we reported a case of asynchronous GCTs across multiple body sites. The pathologic findings were consistent with benign GCTs. NGS of four of the GCTs identified a rarely reported activating *PIK3CA* p.H1047R subclonal mutation in one tumor. A *PIK3CA* mutation is usually a transforming event and its clinical significance in the GCTs requires further evaluation. Genetic testing should be considered in patients with multiple GCTs as some of them with associated syndromes are predisposed to other malignancies.

## Data Availability

The data used to support the findings of this study are available from the corresponding author upon request.
